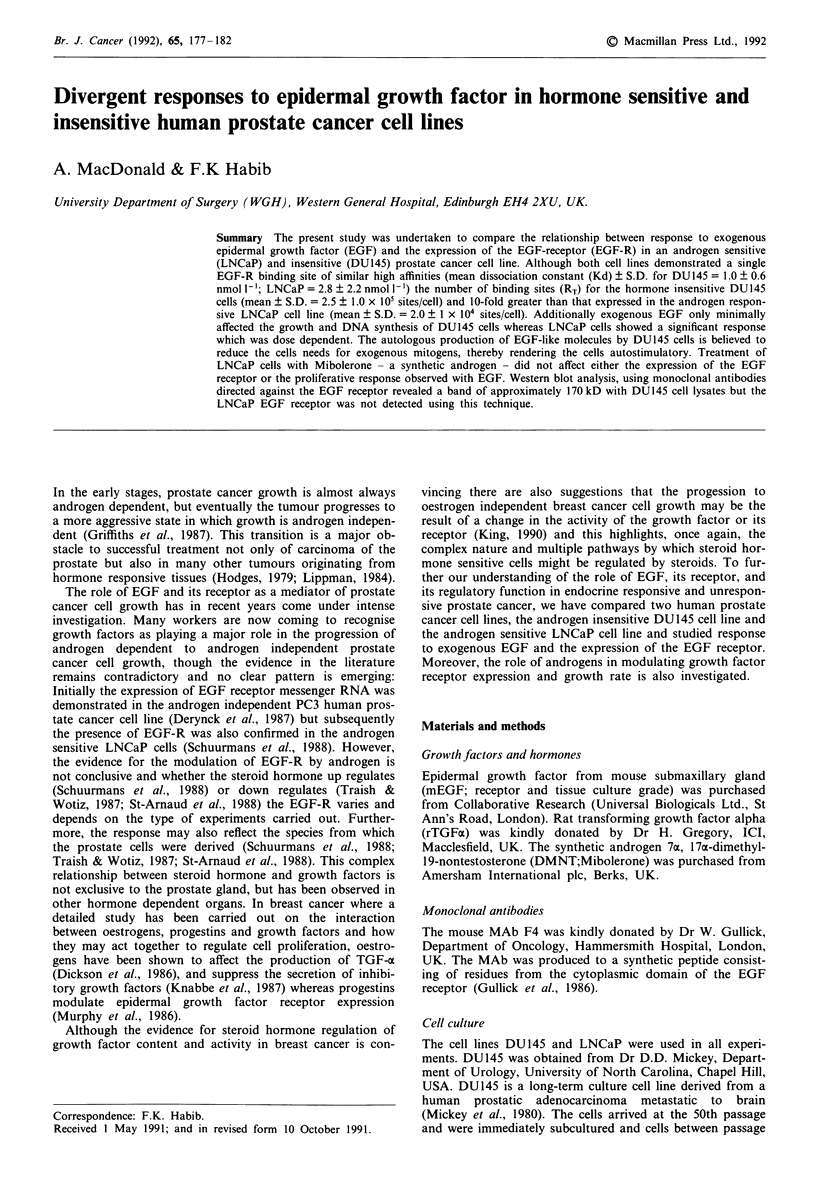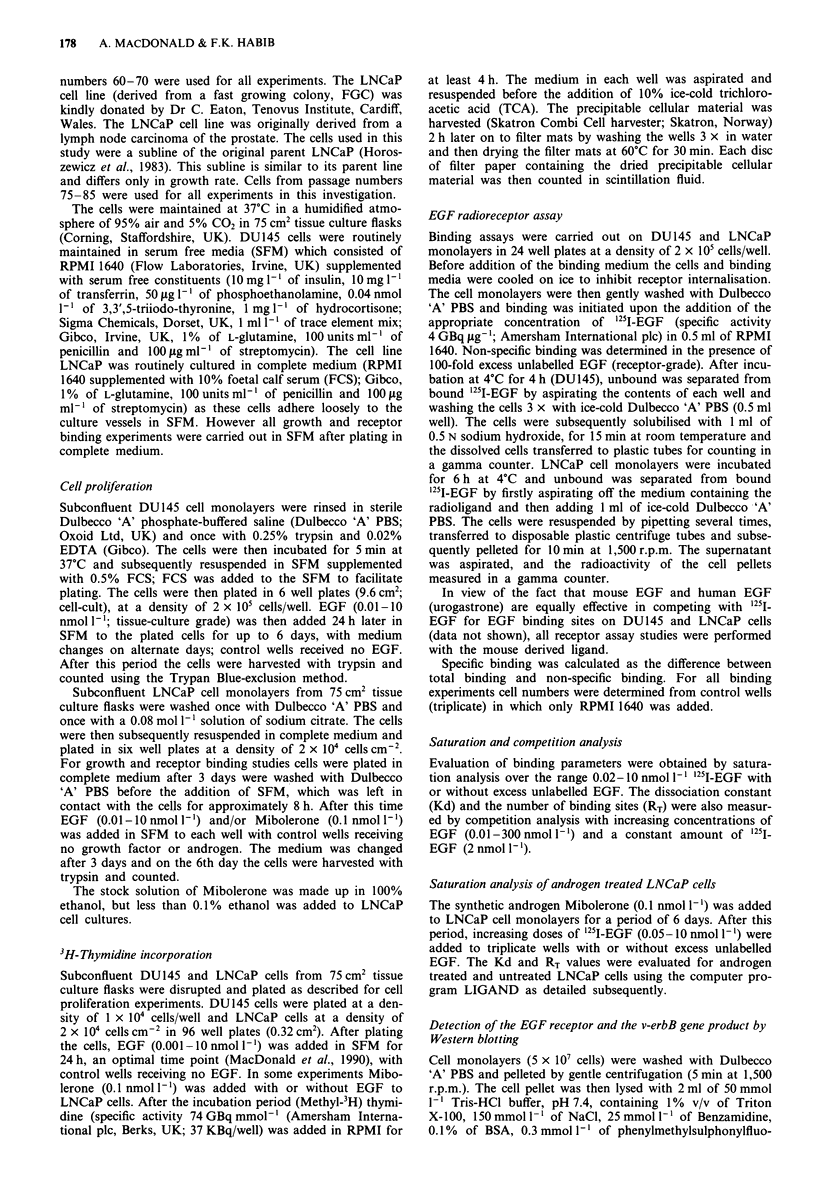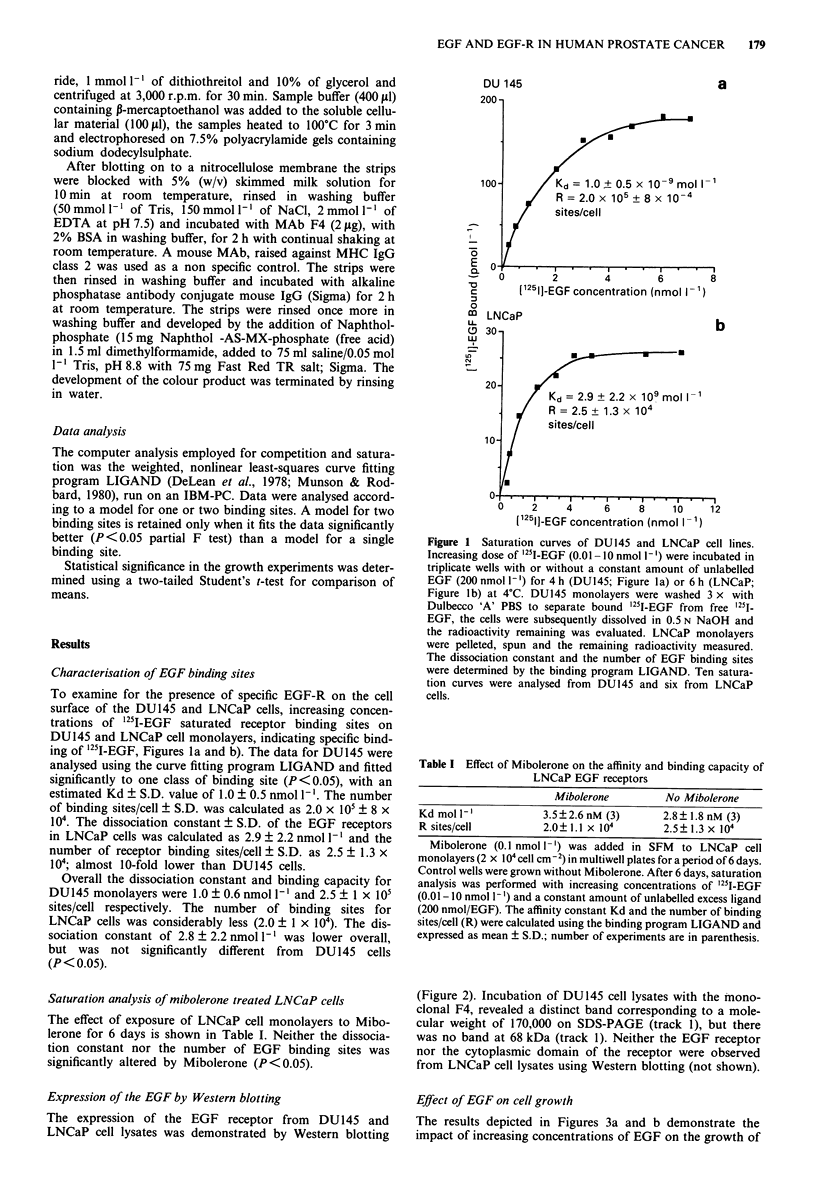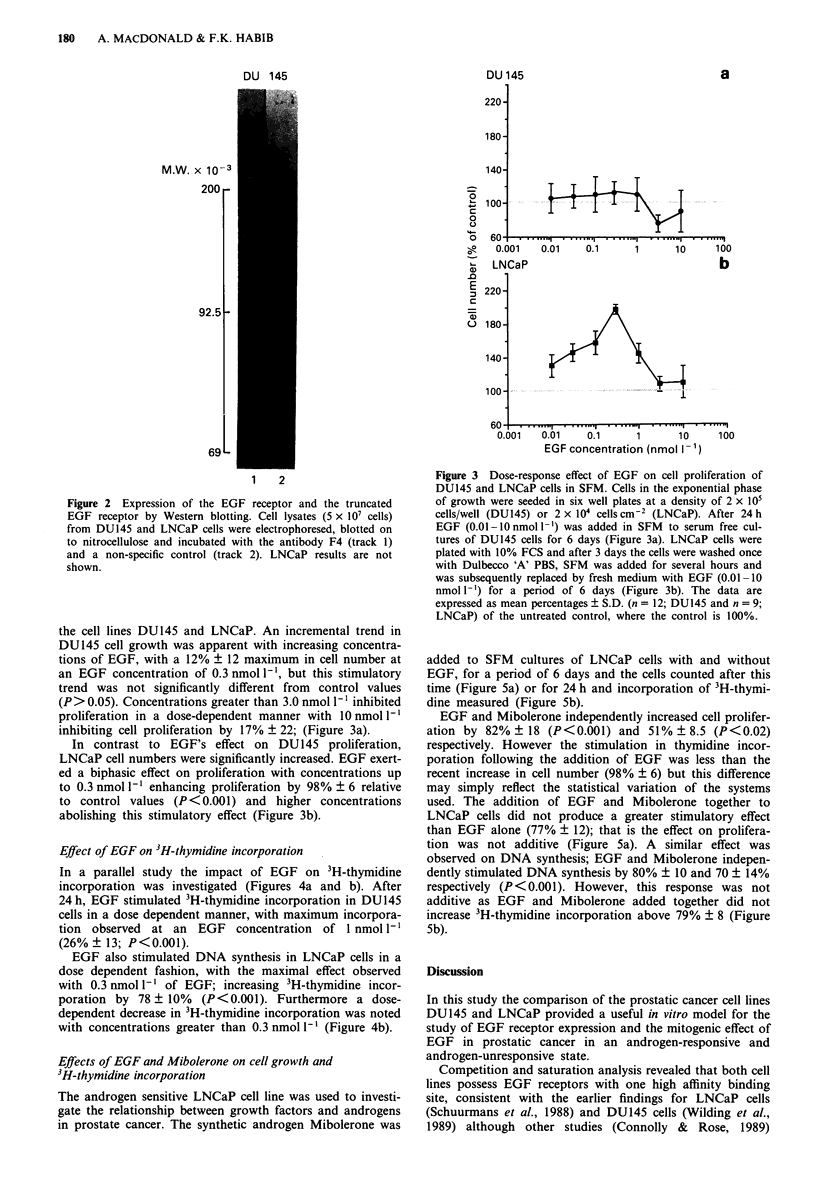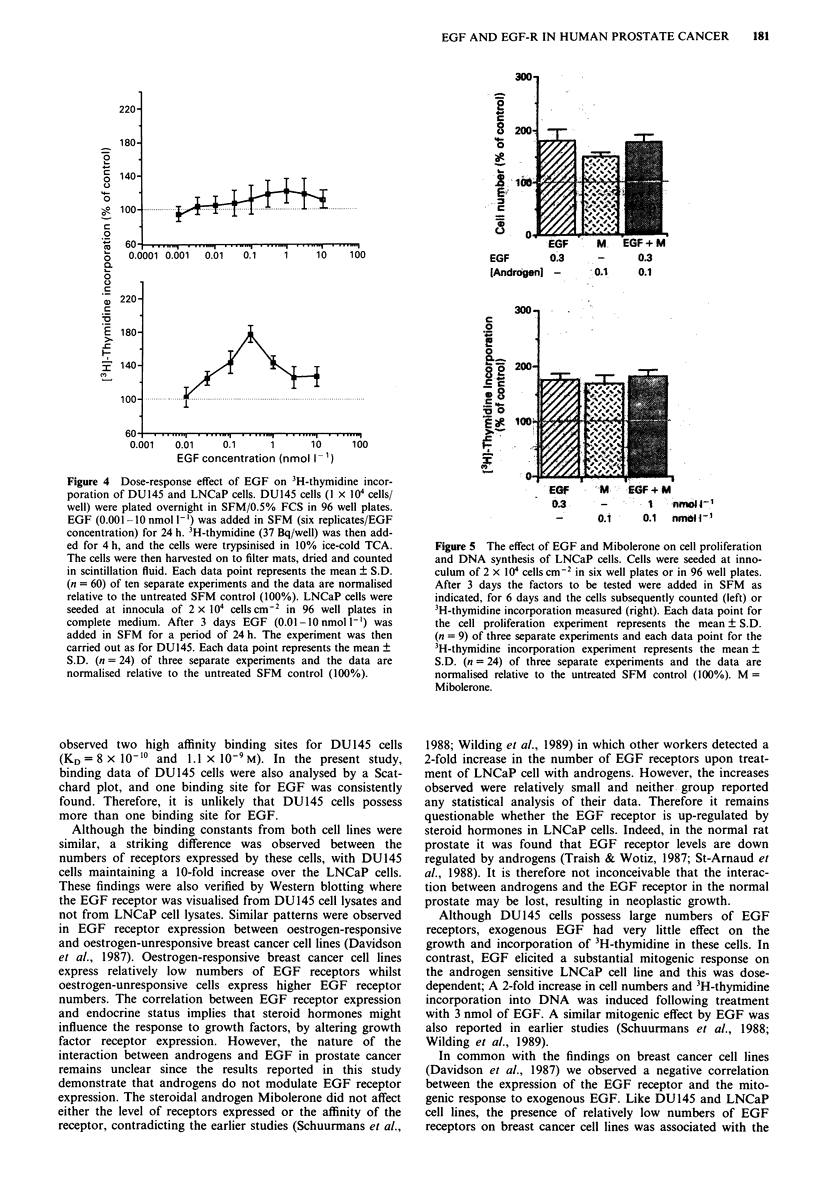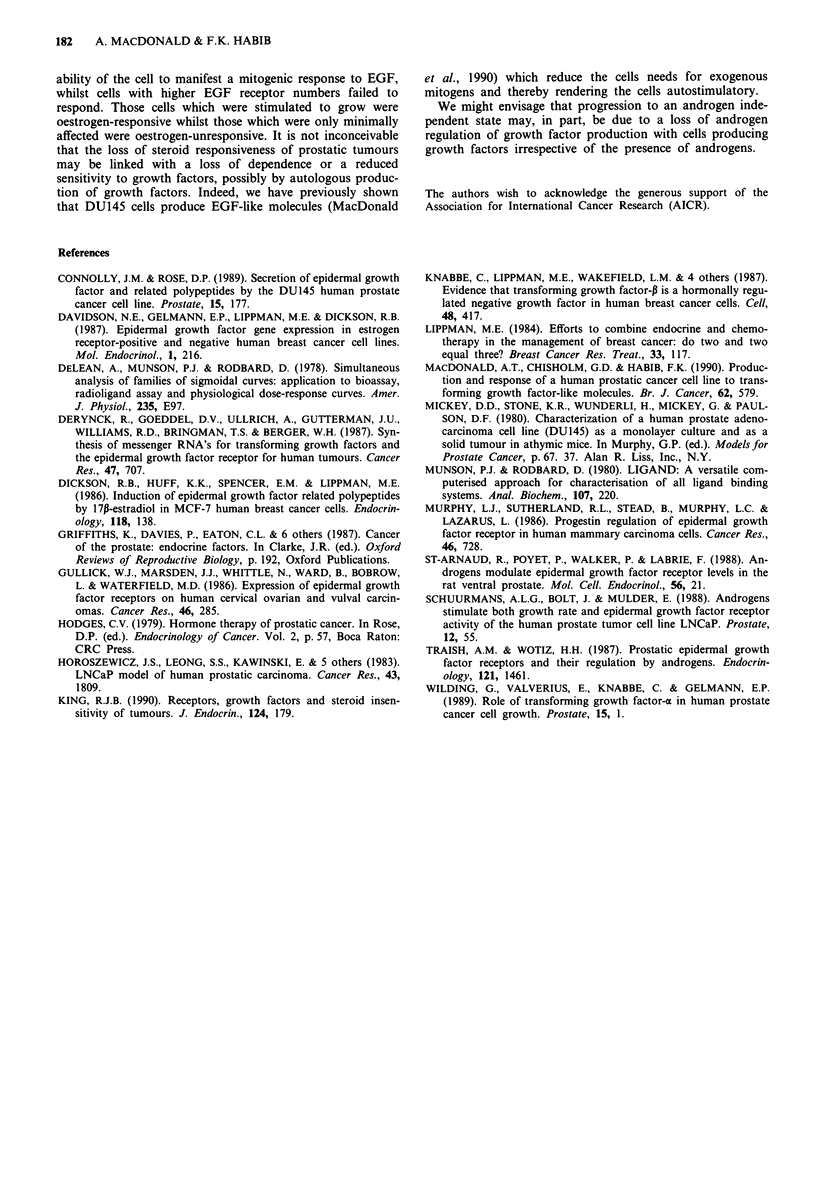# Divergent responses to epidermal growth factor in hormone sensitive and insensitive human prostate cancer cell lines.

**DOI:** 10.1038/bjc.1992.37

**Published:** 1992-02

**Authors:** A. MacDonald, F. K. Habib

**Affiliations:** University Department of Surgery, Western General Hospital, Edinburgh, UK.

## Abstract

**Images:**


Br. J. Cancer (1992), 65, 177-182 © Macmillan Press Ltd., 1992

### Divergent responses to epidermal growth factor in hormone sensitive and insensitive human prostate cancer cell lines

A. MacDonald & F.K Habib

*University Department of Surgery (WGH), Western General Hospital, Edinburgh EH4 2XU, UK.*

**Summary** The present study was undertaken to compare the relationship between response to exogenous epidermal growth factor (EGF) and the expression of the EGF-receptor (EGF-R) in an androgen sensitive (LNCaP) and insensitive (DU145) prostate cancer cell line. Although both cell lines demonstrated a single EGF-R binding site of similar high affinities (mean dissociation constant (Kd) ± S.D. for DU145 = 1.0 ± 0.6 nmol $l^{-1}$; LNCaP = 2.8 ± 2.2 nmol $l^{-1}$) the number of binding sites ($R_T$) for the hormone insensitive DU145 cells (mean ± S.D. = 2.5 ± 1.0 × $10^5$ sites/cell) and 10-fold greater than that expressed in the androgen responsive LNCaP cell line (mean ± S.D. = 2.0 ± 1 × $10^4$ sites/cell). Additionally exogenous EGF only minimally affected the growth and DNA synthesis of DU145 cells whereas LNCaP cells showed a significant response which was dose dependent. The autologous production of EGF-like molecules by DU145 cells is believed to reduce the cells needs for exogenous mitogens, thereby rendering the cells autostimulatory. Treatment of LNCaP cells with Mibolerone – a synthetic androgen – did not affect either the expression of the EGF receptor or the proliferative response observed with EGF. Western blot analysis, using monoclonal antibodies directed against the EGF receptor revealed a band of approximately 170 kD with DU145 cell lysates but the LNCaP EGF receptor was not detected using this technique.

In the early stages, prostate cancer growth is almost always androgen dependent, but eventually the tumour progresses to a more aggressive state in which growth is androgen independent (Griffiths *et al.*, 1987). This transition is a major obstacle to successful treatment not only of carcinoma of the prostate but also in many other tumours originating from hormone responsive tissues (Hodges, 1979; Lippman, 1984).

The role of EGF and its receptor as a mediator of prostate cancer cell growth has in recent years come under intense investigation. Many workers are now coming to recognise growth factors as playing a major role in the progression of androgen dependent to androgen independent prostate cancer cell growth, though the evidence in the literature remains contradictory and no clear pattern is emerging: Initially the expression of EGF receptor messenger RNA was demonstrated in the androgen independent PC3 human prostate cancer cell line (Derynck *et al.*, 1987) but subsequently the presence of EGF-R was also confirmed in the androgen sensitive LNCaP cells (Schuurmans *et al.*, 1988). However, the evidence for the modulation of EGF-R by androgen is not conclusive and whether the steroid hormone up regulates (Schuurmans *et al.*, 1988) or down regulates (Traish & Wotiz, 1987; St-Arnaud *et al.*, 1988) the EGF-R varies and depends on the type of experiments carried out. Furthermore, the response may also reflect the species from which the prostate cells were derived (Schuurmans *et al.*, 1988; Traish & Wotiz, 1987; St-Arnaud *et al.*, 1988). This complex relationship between steroid hormone and growth factors is not exclusive to the prostate gland, but has been observed in other hormone dependent organs. In breast cancer where a detailed study has been carried out on the interaction between oestrogens, progestins and growth factors and how they may act together to regulate cell proliferation, oestrogens have been shown to affect the production of TGF-α (Dickson *et al.*, 1986), and suppress the secretion of inhibitory growth factors (Knabbe *et al.*, 1987) whereas progestins modulate epidermal growth factor receptor expression (Murphy *et al.*, 1986).

Although the evidence for steroid hormone regulation of growth factor content and activity in breast cancer is convincing there are also suggestions that the progession to oestrogen independent breast cancer cell growth may be the result of a change in the activity of the growth factor or its receptor (King, 1990) and this highlights, once again, the complex nature and multiple pathways by which steroid hormone sensitive cells might be regulated by steroids. To further our understanding of the role of EGF, its receptor, and its regulatory function in endocrine responsive and unresponsive prostate cancer, we have compared two human prostate cancer cell lines, the androgen insensitive DU145 cell line and the androgen sensitive LNCaP cell line and studied response to exogenous EGF and the expression of the EGF receptor. Moreover, the role of androgens in modulating growth factor receptor expression and growth rate is also investigated.

### Materials and methods

### *Growth factors and hormones*

Epidermal growth factor from mouse submaxillary gland (mEGF; receptor and tissue culture grade) was purchased from Collaborative Research (Universal Biologicals Ltd., St Ann's Road, London). Rat transforming growth factor alpha (rTGFα) was kindly donated by Dr H. Gregory, ICI, Macclesfield, UK. The synthetic androgen 7α, 17α-dimethyl-19-nontestosterone (DMNT;Mibolerone) was purchased from Amersham International plc, Berks, UK.

### *Monoclonal antibodies*

The mouse MAb F4 was kindly donated by Dr W. Gullick, Department of Oncology, Hammersmith Hospital, London, UK. The MAb was produced to a synthetic peptide consisting of residues from the cytoplasmic domain of the EGF receptor (Gullick *et al.*, 1986).

### *Cell culture*

The cell lines DU145 and LNCaP were used in all experiments. DU145 was obtained from Dr D.D. Mickey, Department of Urology, University of North Carolina, Chapel Hill, USA. DU145 is a long-term culture cell line derived from a human prostatic adenocarcinoma metastatic to brain (Mickey *et al.*, 1980). The cells arrived at the 50th passage and were immediately subcultured and cells between passage

Correspondence: F.K. Habib.
Received 1 May 1991; and in revised form 10 October 1991.

numbers 60–70 were used for all experiments. The LNCaP cell line (derived from a fast growing colony, FGC) was kindly donated by Dr C. Eaton, Tenovus Institute, Cardiff, Wales. The LNCaP cell line was originally derived from a lymph node carcinoma of the prostate. The cells used in this study were a subline of the original parent LNCaP (Horoszewicz *et al.*, 1983). This subline is similar to its parent line and differs only in growth rate. Cells from passage numbers 75–85 were used for all experiments in this investigation.

The cells were maintained at 37°C in a humidified atmosphere of 95% air and 5% $CO_2$ in 75 $cm^2$ tissue culture flasks (Corning, Staffordshire, UK). DU145 cells were routinely maintained in serum free media (SFM) which consisted of RPMI 1640 (Flow Laboratories, Irvine, UK) supplemented with serum free constituents (10 mg $l^{-1}$ of insulin, 10 mg $l^{-1}$ of transferrin, 50 μg $l^{-1}$ of phosphoethanolamine, 0.04 nmol $l^{-1}$ of 3,3′,5-triiodo-thyronine, 1 mg $l^{-1}$ of hydrocortisone; Sigma Chemicals, Dorset, UK, 1 ml $l^{-1}$ of trace element mix; Gibco, Irvine, UK, 1% of L-glutamine, 100 units $ml^{-1}$ of penicillin and 100 μg $ml^{-1}$ of streptomycin). The cell line LNCaP was routinely cultured in complete medium (RPMI 1640 supplemented with 10% foetal calf serum (FCS); Gibco, 1% of L-glutamine, 100 units $ml^{-1}$ of penicillin and 100 μg $ml^{-1}$ of streptomycin) as these cells adhere loosely to the culture vessels in SFM. However all growth and receptor binding experiments were carried out in SFM after plating in complete medium.

*Cell proliferation*

Subconfluent DU145 cell monolayers were rinsed in sterile Dulbecco 'A' phosphate-buffered saline (Dulbecco 'A' PBS; Oxoid Ltd, UK) and once with 0.25% trypsin and 0.02% EDTA (Gibco). The cells were then incubated for 5 min at 37°C and subsequently resuspended in SFM supplemented with 0.5% FCS; FCS was added to the SFM to facilitate plating. The cells were then plated in 6 well plates (9.6 $cm^2$; cell-cult), at a density of $2 \times 10^5$ cells/well. EGF (0.01–10 nmol $l^{-1}$; tissue-culture grade) was then added 24 h later in SFM to the plated cells for up to 6 days, with medium changes on alternate days; control wells received no EGF. After this period the cells were harvested with trypsin and counted using the Trypan Blue-exclusion method.

Subconfluent LNCaP cell monolayers from 75 $cm^2$ tissue culture flasks were washed once with Dulbecco 'A' PBS and once with a 0.08 mol $l^{-1}$ solution of sodium citrate. The cells were then subsequently resuspended in complete medium and plated in six well plates at a density of $2 \times 10^4$ cells $cm^{-2}$. For growth and receptor binding studies cells were plated in complete medium after 3 days were washed with Dulbecco 'A' PBS before the addition of SFM, which was left in contact with the cells for approximately 8 h. After this time EGF (0.01–10 nmol $l^{-1}$) and/or Mibolerone (0.1 nmol $l^{-1}$) was added in SFM to each well with control wells receiving no growth factor or androgen. The medium was changed after 3 days and on the 6th day the cells were harvested with trypsin and counted.

The stock solution of Mibolerone was made up in 100% ethanol, but less than 0.1% ethanol was added to LNCaP cell cultures.

*$^3$H-Thymidine incorporation*

Subconfluent DU145 and LNCaP cells from 75 $cm^2$ tissue culture flasks were disrupted and plated as described for cell proliferation experiments. DU145 cells were plated at a density of $1 \times 10^4$ cells/well and LNCaP cells at a density of $2 \times 10^4$ cells $cm^{-2}$ in 96 well plates (0.32 $cm^2$). After plating the cells, EGF (0.001–10 nmol $l^{-1}$) was added in SFM for 24 h, an optimal time point (MacDonald *et al.*, 1990), with control wells receiving no EGF. In some experiments Mibolerone (0.1 nmol $l^{-1}$) was added with or without EGF to LNCaP cells. After the incubation period (Methyl-$^3$H) thymidine (specific activity 74 GBq $mmol^{-1}$ (Amersham International plc, Berks, UK; 37 KBq/well) was added in RPMI for at least 4 h. The medium in each well was aspirated and resuspended before the addition of 10% ice-cold trichloroacetic acid (TCA). The precipitable cellular material was harvested (Skatron Combi Cell harvester; Skatron, Norway) 2 h later on to filter mats by washing the wells 3 × in water and then drying the filter mats at 60°C for 30 min. Each disc of filter paper containing the dried precipitable cellular material was then counted in scintillation fluid.

*EGF radioreceptor assay*

Binding assays were carried out on DU145 and LNCaP monolayers in 24 well plates at a density of $2 \times 10^5$ cells/well. Before addition of the binding medium the cells and binding media were cooled on ice to inhibit receptor internalisation. The cell monolayers were then gently washed with Dulbecco 'A' PBS and binding was initiated upon the addition of the appropriate concentration of $^{125}$I-EGF (specific activity 4 GBq $\mu g^{-1}$; Amersham International plc) in 0.5 ml of RPMI 1640. Non-specific binding was determined in the presence of 100-fold excess unlabelled EGF (receptor-grade). After incubation at 4°C for 4 h (DU145), unbound was separated from bound $^{125}$I-EGF by aspirating the contents of each well and washing the cells 3 × with ice-cold Dulbecco 'A' PBS (0.5 ml well). The cells were subsequently solubilised with 1 ml of 0.5 N sodium hydroxide, for 15 min at room temperature and the dissolved cells transferred to plastic tubes for counting in a gamma counter. LNCaP cell monolayers were incubated for 6 h at 4°C and unbound was separated from bound $^{125}$I-EGF by firstly aspirating off the medium containing the radioligand and then adding 1 ml of ice-cold Dulbecco 'A' PBS. The cells were resuspended by pipetting several times, transferred to disposable plastic centrifuge tubes and subsequently pelleted for 10 min at 1,500 r.p.m. The supernatant was aspirated, and the radioactivity of the cell pellets measured in a gamma counter.

In view of the fact that mouse EGF and human EGF (urogastrone) are equally effective in competing with $^{125}$I-EGF for EGF binding sites on DU145 and LNCaP cells (data not shown), all receptor assay studies were performed with the mouse derived ligand.

Specific binding was calculated as the difference between total binding and non-specific binding. For all binding experiments cell numbers were determined from control wells (triplicate) in which only RPMI 1640 was added.

*Saturation and competition analysis*

Evaluation of binding parameters were obtained by saturation analysis over the range 0.02–10 nmol $l^{-1}$ $^{125}$I-EGF with or without excess unlabelled EGF. The dissociation constant (Kd) and the number of binding sites ($R_T$) were also measured by competition analysis with increasing concentrations of EGF (0.01–300 nmol $l^{-1}$) and a constant amount of $^{125}$I-EGF (2 nmol $l^{-1}$).

*Saturation analysis of androgen treated LNCaP cells*

The synthetic androgen Mibolerone (0.1 nmol $l^{-1}$) was added to LNCaP cell monolayers for a period of 6 days. After this period, increasing doses of $^{125}$I-EGF (0.05–10 nmol $l^{-1}$) were added to triplicate wells with or without excess unlabelled EGF. The Kd and $R_T$ values were evaluated for androgen treated and untreated LNCaP cells using the computer program LIGAND as detailed subsequently.

*Detection of the EGF receptor and the v-erbB gene product by Western blotting*

Cell monolayers ($5 \times 10^7$ cells) were washed with Dulbecco 'A' PBS and pelleted by gentle centrifugation (5 min at 1,500 r.p.m.). The cell pellet was then lysed with 2 ml of 50 mmol $l^{-1}$ Tris-HCl buffer, pH 7.4, containing 1% v/v of Triton X-100, 150 mmol $l^{-1}$ of NaCl, 25 mmol $l^{-1}$ of Benzamidine, 0.1% of BSA, 0.3 mmol $l^{-1}$ of phenylmethylsulphonylfluoride, 1 mmol $l^{-1}$ of dithiothreitol and 10% of glycerol and centrifuged at 3,000 r.p.m. for 30 min. Sample buffer (400 µl) containing β-mercaptoethanol was added to the soluble cellular material (100 µl), the samples heated to 100°C for 3 min and electrophoresed on 7.5% polyacrylamide gels containing sodium dodecylsulphate.

After blotting on to a nitrocellulose membrane the strips were blocked with 5% (w/v) skimmed milk solution for 10 min at room temperature, rinsed in washing buffer (50 mmol $l^{-1}$ of Tris, 150 mmol $l^{-1}$ of NaCl, 2 mmol $l^{-1}$ of EDTA at pH 7.5) and incubated with MAb F4 (2 µg), with 2% BSA in washing buffer, for 2 h with continual shaking at room temperature. A mouse MAb, raised against MHC IgG class 2 was used as a non specific control. The strips were then rinsed in washing buffer and incubated with alkaline phosphatase antibody conjugate mouse IgG (Sigma) for 2 h at room temperature. The strips were rinsed once more in washing buffer and developed by the addition of Naphthol-phosphate (15 mg Naphthol -AS-MX-phosphate (free acid) in 1.5 ml dimethylformamide, added to 75 ml saline/0.05 mol $l^{-1}$ Tris, pH 8.8 with 75 mg Fast Red TR salt; Sigma. The development of the colour product was terminated by rinsing in water.

*Data analysis*

The computer analysis employed for competition and saturation was the weighted, nonlinear least-squares curve fitting program LIGAND (DeLean *et al.*, 1978; Munson & Rodbard, 1980), run on an IBM-PC. Data were analysed according to a model for one or two binding sites. A model for two binding sites is retained only when it fits the data significantly better ($P<0.05$ partial F test) than a model for a single binding site.

Statistical significance in the growth experiments was determined using a two-tailed Student's *t*-test for comparison of means.

### Results

*Characterisation of EGF binding sites*

To examine for the presence of specific EGF-R on the cell surface of the DU145 and LNCaP cells, increasing concentrations of $^{125}$I-EGF saturated receptor binding sites on DU145 and LNCaP cell monolayers, indicating specific binding of $^{125}$I-EGF, Figures 1a and b). The data for DU145 were analysed using the curve fitting program LIGAND and fitted significantly to one class of binding site ($P<0.05$), with an estimated Kd ± S.D. value of 1.0 ± 0.5 nmol $l^{-1}$. The number of binding sites/cell ± S.D. was calculated as 2.0 × $10^5$ ± 8 × $10^4$. The dissociation constant ± S.D. of the EGF receptors in LNCaP cells was calculated as 2.9 ± 2.2 nmol $l^{-1}$ and the number of receptor binding sites/cell ± S.D. as 2.5 ± 1.3 × $10^4$; almost 10-fold lower than DU145 cells.

Overall the dissociation constant and binding capacity for DU145 monolayers were 1.0 ± 0.6 nmol $l^{-1}$ and 2.5 ± 1 × $10^5$ sites/cell respectively. The number of binding sites for LNCaP cells was considerably less (2.0 ± 1 × $10^4$). The dissociation constant of 2.8 ± 2.2 nmol $l^{-1}$ was lower overall, but was not significantly different from DU145 cells ($P<0.05$).

*Saturation analysis of mibolerone treated LNCaP cells*

The effect of exposure of LNCaP cell monolayers to Mibolerone for 6 days is shown in Table I. Neither the dissociation constant nor the number of EGF binding sites was significantly altered by Mibolerone ($P<0.05$).

*Expression of the EGF by Western blotting*

The expression of the EGF receptor from DU145 and LNCaP cell lysates was demonstrated by Western blotting (Figure 2). Incubation of DU145 cell lysates with the monoclonal F4, revealed a distinct band corresponding to a molecular weight of 170,000 on SDS-PAGE (track 1), but there was no band at 68 kDa (track 1). Neither the EGF receptor nor the cytoplasmic domain of the receptor were observed from LNCaP cell lysates using Western blotting (not shown).

**Figure 1** Saturation curves of DU145 and LNCaP cell lines. Increasing dose of $^{125}$I-EGF (0.01–10 nmol $l^{-1}$) were incubated in triplicate wells with or without a constant amount of unlabelled EGF (200 nmol $l^{-1}$) for 4 h (DU145; Figure 1a) or 6 h (LNCaP; Figure 1b) at 4°C. DU145 monolayers were washed 3 × with Dulbecco 'A' PBS to separate bound $^{125}$I-EGF from free $^{125}$I-EGF, the cells were subsequently dissolved in 0.5 N NaOH and the radioactivity remaining was evaluated. LNCaP monolayers were pelleted, spun and the remaining radioactivity measured. The dissociation constant and the number of EGF binding sites were determined by the binding program LIGAND. Ten saturation curves were analysed from DU145 and six from LNCaP cells.

**Table I** Effect of Mibolerone on the affinity and binding capacity of LNCaP EGF receptors

| | *Mibolerone* | *No Mibolerone* |
|---|---|---|
| Kd mol $l^{-1}$ | 3.5±2.6 nM (3) | 2.8±1.8 nM (3) |
| R sites/cell | 2.0±1.1 × $10^4$ | 2.5±1.3 × $10^4$ |

Mibolerone (0.1 nmol $l^{-1}$) was added in SFM to LNCaP cell monolayers (2 × $10^4$ cell $cm^{-2}$) in multiwell plates for a period of 6 days. Control wells were grown without Mibolerone. After 6 days, saturation analysis was performed with increasing concentrations of $^{125}$I-EGF (0.01–10 nmol $l^{-1}$) and a constant amount of unlabelled excess ligand (200 nmol/EGF). The affinity constant Kd and the number of binding sites/cell (R) were calculated using the binding program LIGAND and expressed as mean ± S.D.; number of experiments are in parenthesis.

*Effect of EGF on cell growth*

The results depicted in Figures 3a and b demonstrate the impact of increasing concentrations of EGF on the growth of

**Figure 2** Expression of the EGF receptor and the truncated EGF receptor by Western blotting. Cell lysates ($5 \times 10^7$ cells) from DU145 and LNCaP cells were electrophoresed, blotted on to nitrocellulose and incubated with the antibody F4 (track 1) and a non-specific control (track 2). LNCaP results are not shown.

**Figure 3** Dose-response effect of EGF on cell proliferation of DU145 and LNCaP cells in SFM. Cells in the exponential phase of growth were seeded in six well plates at a density of $2 \times 10^5$ cells/well (DU145) or $2 \times 10^4$ cells cm$^{-2}$ (LNCaP). After 24 h EGF (0.01–10 nmol l$^{-1}$) was added in SFM to serum free cultures of DU145 cells for 6 days (Figure 3a). LNCaP cells were plated with 10% FCS and after 3 days the cells were washed once with Dulbecco 'A' PBS, SFM was added for several hours and was subsequently replaced by fresh medium with EGF (0.01–10 nmol l$^{-1}$) for a period of 6 days (Figure 3b). The data are expressed as mean percentages ± S.D. ($n = 12$; DU145 and $n = 9$; LNCaP) of the untreated control, where the control is 100%.

the cell lines DU145 and LNCaP. An incremental trend in DU145 cell growth was apparent with increasing concentrations of EGF, with a 12% ± 12 maximum in cell number at an EGF concentration of 0.3 nmol l$^{-1}$, but this stimulatory trend was not significantly different from control values ($P > 0.05$). Concentrations greater than 3.0 nmol l$^{-1}$ inhibited proliferation in a dose-dependent manner with 10 nmol l$^{-1}$ inhibiting cell proliferation by 17% ± 22; (Figure 3a).

In contrast to EGF's effect on DU145 proliferation, LNCaP cell numbers were significantly increased. EGF exerted a biphasic effect on proliferation with concentrations up to 0.3 nmol l$^{-1}$ enhancing proliferation by 98% ± 6 relative to control values ($P < 0.001$) and higher concentrations abolishing this stimulatory effect (Figure 3b).

### *Effect of EGF on $^3$H-thymidine incorporation*

In a parallel study the impact of EGF on $^3$H-thymidine incorporation was investigated (Figures 4a and b). After 24 h, EGF stimulated $^3$H-thymidine incorporation in DU145 cells in a dose dependent manner, with maximum incorporation observed at an EGF concentration of 1 nmol l$^{-1}$ (26% ± 13; $P < 0.001$).

EGF also stimulated DNA synthesis in LNCaP cells in a dose dependent fashion, with the maximal effect observed with 0.3 nmol l$^{-1}$ of EGF; increasing $^3$H-thymidine incorporation by 78 ± 10% ($P < 0.001$). Furthermore a dose-dependent decrease in $^3$H-thymidine incorporation was noted with concentrations greater than 0.3 nmol l$^{-1}$ (Figure 4b).

### *Effects of EGF and Mibolerone on cell growth and $^3$H-thymidine incorporation*

The androgen sensitive LNCaP cell line was used to investigate the relationship between growth factors and androgens in prostate cancer. The synthetic androgen Mibolerone was added to SFM cultures of LNCaP cells with and without EGF, for a period of 6 days and the cells counted after this time (Figure 5a) or for 24 h and incorporation of $^3$H-thymidine measured (Figure 5b).

EGF and Mibolerone independently increased cell proliferation by 82% ± 18 ($P < 0.001$) and 51% ± 8.5 ($P < 0.02$) respectively. However the stimulation in thymidine incorporation following the addition of EGF was less than the recent increase in cell number (98% ± 6) but this difference may simply reflect the statistical variation of the systems used. The addition of EGF and Mibolerone together to LNCaP cells did not produce a greater stimulatory effect than EGF alone (77% ± 12); that is the effect on proliferation was not additive (Figure 5a). A similar effect was observed on DNA synthesis; EGF and Mibolerone independently stimulated DNA synthesis by 80% ± 10 and 70 ± 14% respectively ($P < 0.001$). However, this response was not additive as EGF and Mibolerone added together did not increase $^3$H-thymidine incorporation above 79% ± 8 (Figure 5b).

### Discussion

In this study the comparison of the prostatic cancer cell lines DU145 and LNCaP provided a useful *in vitro* model for the study of EGF receptor expression and the mitogenic effect of EGF in prostatic cancer in an androgen-responsive and androgen-unresponsive state.

Competition and saturation analysis revealed that both cell lines possess EGF receptors with one high affinity binding site, consistent with the earlier findings for LNCaP cells (Schuurmans *et al.*, 1988) and DU145 cells (Wilding *et al.*, 1989) although other studies (Connolly & Rose, 1989)

**Figure 4** Dose-response effect of EGF on $^{3}$H-thymidine incorporation of DU145 and LNCaP cells. DU145 cells (1 × 10$^{4}$ cells/well) were plated overnight in SFM/0.5% FCS in 96 well plates. EGF (0.001–10 nmol l$^{-1}$) was added in SFM (six replicates/EGF concentration) for 24 h. $^{3}$H-thymidine (37 Bq/well) was then added for 4 h, and the cells were trypsinised in 10% ice-cold TCA. The cells were then harvested on to filter mats, dried and counted in scintillation fluid. Each data point represents the mean ± S.D. ($n$ = 60) of ten separate experiments and the data are normalised relative to the untreated SFM control (100%). LNCaP cells were seeded at innocula of 2 × 10$^{4}$ cells cm$^{-2}$ in 96 well plates in complete medium. After 3 days EGF (0.01–10 nmol l$^{-1}$) was added in SFM for a period of 24 h. The experiment was then carried out as for DU145. Each data point represents the mean ± S.D. ($n$ = 24) of three separate experiments and the data are normalised relative to the untreated SFM control (100%).

**Figure 5** The effect of EGF and Mibolerone on cell proliferation and DNA synthesis of LNCaP cells. Cells were seeded at innoculum of 2 × 10$^{4}$ cells cm$^{-2}$ in six well plates or in 96 well plates. After 3 days the factors to be tested were added in SFM as indicated, for 6 days and the cells subsequently counted (left) or $^{3}$H-thymidine incorporation measured (right). Each data point for the cell proliferation experiment represents the mean ± S.D. ($n$ = 9) of three separate experiments and each data point for the $^{3}$H-thymidine incorporation experiment represents the mean ± S.D. ($n$ = 24) of three separate experiments and the data are normalised relative to the untreated SFM control (100%). M = Mibolerone.

observed two high affinity binding sites for DU145 cells ($K_D$ = 8 × 10$^{-10}$ and 1.1 × 10$^{-9}$ M). In the present study, binding data of DU145 cells were also analysed by a Scatchard plot, and one binding site for EGF was consistently found. Therefore, it is unlikely that DU145 cells possess more than one binding site for EGF.

Although the binding constants from both cell lines were similar, a striking difference was observed between the numbers of receptors expressed by these cells, with DU145 cells maintaining a 10-fold increase over the LNCaP cells. These findings were also verified by Western blotting where the EGF receptor was visualised from DU145 cell lysates and not from LNCaP cell lysates. Similar patterns were observed in EGF receptor expression between oestrogen-responsive and oestrogen-unresponsive breast cancer cell lines (Davidson *et al.*, 1987). Oestrogen-responsive breast cancer cell lines express relatively low numbers of EGF receptors whilst oestrogen-unresponsive cells express higher EGF receptor numbers. The correlation between EGF receptor expression and endocrine status implies that steroid hormones might influence the response to growth factors, by altering growth factor receptor expression. However, the nature of the interaction between androgens and EGF in prostate cancer remains unclear since the results reported in this study demonstrate that androgens do not modulate EGF receptor expression. The steroidal androgen Mibolerone did not affect either the level of receptors expressed or the affinity of the receptor, contradicting the earlier studies (Schuurmans *et al.*, 1988; Wilding *et al.*, 1989) in which other workers detected a 2-fold increase in the number of EGF receptors upon treatment of LNCaP cell with androgens. However, the increases observed were relatively small and neither group reported any statistical analysis of their data. Therefore it remains questionable whether the EGF receptor is up-regulated by steroid hormones in LNCaP cells. Indeed, in the normal rat prostate it was found that EGF receptor levels are down regulated by androgens (Traish & Wotiz, 1987; St-Arnaud *et al.*, 1988). It is therefore not inconceivable that the interaction between androgens and the EGF receptor in the normal prostate may be lost, resulting in neoplastic growth.

Although DU145 cells possess large numbers of EGF receptors, exogenous EGF had very little effect on the growth and incorporation of $^{3}$H-thymidine in these cells. In contrast, EGF elicited a substantial mitogenic response on the androgen sensitive LNCaP cell line and this was dose-dependent; A 2-fold increase in cell numbers and $^{3}$H-thymidine incorporation into DNA was induced following treatment with 3 nmol of EGF. A similar mitogenic effect by EGF was also reported in earlier studies (Schuurmans *et al.*, 1988; Wilding *et al.*, 1989).

In common with the findings on breast cancer cell lines (Davidson *et al.*, 1987) we observed a negative correlation between the expression of the EGF receptor and the mitogenic response to exogenous EGF. Like DU145 and LNCaP cell lines, the presence of relatively low numbers of EGF receptors on breast cancer cell lines was associated with the ability of the cell to manifest a mitogenic response to EGF, whilst cells with higher EGF receptor numbers failed to respond. Those cells which were stimulated to grow were oestrogen-responsive whilst those which were only minimally affected were oestrogen-unresponsive. It is not inconceivable that the loss of steroid responsiveness of prostatic tumours may be linked with a loss of dependence or a reduced sensitivity to growth factors, possibly by autologous production of growth factors. Indeed, we have previously shown that DU145 cells produce EGF-like molecules (MacDonald *et al.*, 1990) which reduce the cells needs for exogenous mitogens and thereby rendering the cells autostimulatory.

We might envisage that progression to an androgen independent state may, in part, be due to a loss of androgen regulation of growth factor production with cells producing growth factors irrespective of the presence of androgens.

The authors wish to acknowledge the generous support of the Association for International Cancer Research (AICR).

### References

CONNOLLY, J.M. & ROSE, D.P. (1989). Secretion of epidermal growth factor and related polypeptides by the DU145 human prostate cancer cell line. *Prostate*, **15**, 177.

DAVIDSON, N.E., GELMANN, E.P., LIPPMAN, M.E. & DICKSON, R.B. (1987). Epidermal growth factor gene expression in estrogen receptor-positive and negative human breast cancer cell lines. *Mol. Endocrinol.*, **1**, 216.

DELEAN, A., MUNSON, P.J. & RODBARD, D. (1978). Simultaneous analysis of families of sigmoidal curves: application to bioassay, radioligand assay and physiological dose-response curves. *Amer. J. Physiol.*, **235**, E97.

DERYNCK, R., GOEDDEL, D.V., ULLRICH, A., GUTTERMAN, J.U., WILLIAMS, R.D., BRINGMAN, T.S. & BERGER, W.H. (1987). Synthesis of messenger RNA's for transforming growth factors and the epidermal growth factor receptor for human tumours. *Cancer Res.*, **47**, 707.

DICKSON, R.B., HUFF, K.K., SPENCER, E.M. & LIPPMAN, M.E. (1986). Induction of epidermal growth factor related polypeptides by 17β-estradiol in MCF-7 human breast cancer cells. *Endocrinology*, **118**, 138.

GRIFFITHS, K., DAVIES, P., EATON, C.L. & 6 others (1987). Cancer of the prostate: endocrine factors. In Clarke, J.R. (ed.). *Oxford Reviews of Reproductive Biology*, p. 192, Oxford Publications.

GULLICK, W.J., MARSDEN, J.J., WHITTLE, N., WARD, B., BOBROW, L. & WATERFIELD, M.D. (1986). Expression of epidermal growth factor receptors on human cervical ovarian and vulval carcinomas. *Cancer Res.*, **46**, 285.

HODGES, C.V. (1979). Hormone therapy of prostatic cancer. In Rose, D.P. (ed.). *Endocrinology of Cancer*. Vol. 2, p. 57, Boca Raton: CRC Press.

HOROSZEWICZ, J.S., LEONG, S.S., KAWINSKI, E. & 5 others (1983). LNCaP model of human prostatic carcinoma. *Cancer Res.*, **43**, 1809.

KING, R.J.B. (1990). Receptors, growth factors and steroid insensitivity of tumours. *J. Endocrin.*, **124**, 179.

KNABBE, C., LIPPMAN, M.E., WAKEFIELD, L.M. & 4 others (1987). Evidence that transforming growth factor-β is a hormonally regulated negative growth factor in human breast cancer cells. *Cell*, **48**, 417.

LIPPMAN, M.E. (1984). Efforts to combine endocrine and chemotherapy in the management of breast cancer: do two and two equal three? *Breast Cancer Res. Treat.*, **33**, 117.

MACDONALD, A.T., CHISHOLM, G.D. & HABIB, F.K. (1990). Production and response of a human prostatic cancer cell line to transforming growth factor-like molecules. *Br. J. Cancer*, **62**, 579.

MICKEY, D.D., STONE, K.R., WUNDERLI, H., MICKEY, G. & PAULSON, D.F. (1980). Characterization of a human prostate adenocarcinoma cell line (DU145) as a monolayer culture and as a solid tumour in athymic mice. In Murphy, G.P. (ed.). *Models for Prostate Cancer*, p. 67. 37. Alan R. Liss, Inc., N.Y.

MUNSON, P.J. & RODBARD, D. (1980). LIGAND: A versatile computerised approach for characterisation of all ligand binding systems. *Anal. Biochem.*, **107**, 220.

MURPHY, L.J., SUTHERLAND, R.L., STEAD, B., MURPHY, L.C. & LAZARUS, L. (1986). Progestin regulation of epidermal growth factor receptor in human mammary carcinoma cells. *Cancer Res.*, **46**, 728.

ST-ARNAUD, R., POYET, P., WALKER, P. & LABRIE, F. (1988). Androgens modulate epidermal growth factor receptor levels in the rat ventral prostate. *Mol. Cell. Endocrinol.*, **56**, 21.

SCHUURMANS, A.L.G., BOLT, J. & MULDER, E. (1988). Androgens stimulate both growth rate and epidermal growth factor receptor activity of the human prostate tumor cell line LNCaP. *Prostate*, **12**, 55.

TRAISH, A.M. & WOTIZ, H.H. (1987). Prostatic epidermal growth factor receptors and their regulation by androgens. *Endocrinology*, **121**, 1461.

WILDING, G., VALVERIUS, E., KNABBE, C. & GELMANN, E.P. (1989). Role of transforming growth factor-α in human prostate cancer cell growth. *Prostate*, **15**, 1.